# A Review on Progress in QSPR Studies for Surfactants

**DOI:** 10.3390/ijms11031020

**Published:** 2010-03-08

**Authors:** Jiwei Hu, Xiaoyi Zhang, Zhengwu Wang

**Affiliations:** 1 Guizhou Provincial Key Laboratory for Information System of Mountainous Areas and Protection of Ecological Environment, Guizhou Normal University, Guiyang City, Guizhou Province, 550001, China; E-Mail: jwhu@gznu.edu.cn (J.H.); 2 School of Chemistry and Materials Science, Guizhou Normal University, Guiyang City, Guizhou Province, 550001, China; 3 Department of Food Science and Technology, Bor Luh Food Safety Center, Shanghai JiaoTong University, Shanghai, 201101, China

**Keywords:** surfactants, cmc, surface tension, cloud point, biodegradation, QSPR

## Abstract

This paper presents a review on recent progress in quantitative structure-property relationship (QSPR) studies of surfactants and applications of various molecular descriptors. QSPR studies on critical micelle concentration (cmc) and surface tension (γ) of surfactants are introduced. Studies on charge distribution in ionic surfactants by quantum chemical calculations and its effects on the structures and properties of the colloids of surfactants are also reviewed. The trends of QSPR studies on cloud point (for nonionic surfactants), biodegradation potential and some other properties of surfactants are evaluated.

## 1. Introduction

Surfactants are usually amphiphilic organic compounds, meaning they contain both hydrophobic groups (their “tails”) and hydrophilic groups (their “heads”). Therefore, they are soluble in both organic solvents and water. Due to their unique amphiphilic structures, surfactants have been widely used in traditional industries [1]. Furthermore their applications in many fields of science and technology have recently been expanded [2–4]. Preparation of size-controllable nanoparticles by application of micelles and microemulsions as well as of porous materials by application of liquid crystals have been well documented [5,6]. Various membrane structures mainly consisting of amphiphilic molecules can be applied to fields such as photochemical solar energy transformation, molecular recognition, pharmaceutical formulation, targeting and sustained-release, and provision of unique micro-environments for substrates and enzymes and enzyme immobilization [7–10], and even restoration of environmental contamination [11].

On the other hand, close attention has been given to the impact on the environment, especially on soils and waters, caused by the use of large quantities of surfactants [12]. It is especially worth noting that due to their special structures, perfluorooctane sulfonate (PFOS) and perfluorooctanoic acid (PFOA) surfactants are an important class of perfluorinated compound (PFCs) and also a class of emerging persistent organic pollutants (POPs) due to their high chemical stability and slow degradation. Fluorosurfactants are synthetic organofluorine chemical compounds that have multiple fluorine atoms. They can be polyfluorinated or fluorocarbon-based (perfluorinated) [13]. Fluorosurfactants have a fluorinated “tail” and a hydrophilic “head”, and are more effective at lowering the surface tension of water than comparable hydrocarbon surfactants, as they can lower the surface tension of water down to a value half of what is attainable by using hydrocarbon surfactants [14]. Some fluorosurfactants, such as PFOS, are detected in humans and wildlife, and fluorosurfactants tend to concentrate at the liquid-air interface due to the lipophobic nature of fluorocarbons [15]. They are not susceptible to the London dispersion force, the basis for lipophilicity, because the electronegativity of fluorine reduces the polarizability of the surfactants' fluorinated molecular surface. Therefore, the attractive interactions resulting from the “fleeting dipoles” are reduced, in comparison to hydrocarbon surfactants. However, fluorosurfactants are more stable and fit for harsh conditions than hydrocarbon surfactants because of the stability of the carbon–fluorine bond. Likewise, fluorosurfactants can persist in the environment due to their high stability.

Consequently, studies on quantitative structure-property relationships (QSPRs) of surfactants and understanding of the effects of molecular structures on their functions and properties are becoming increasingly important [1,16]. In the processes for study, development and application of surfactants, a broad range of data concerning their properties and activities have been accumulated. Using thermodynamic data and other experimental data, widely applicable and acceptable QSPR models have been established between basic structures and physicochemical properties, applicable functions and some other special properties [17–21]. However, these QSPR models regarding surfactants are generally based only on the summary of a large amount of experimental data, and no detailed studies on their mechanisms of interaction have been performed. At present, QSPR methods based on the studies by Hansch and Free-Wilson [22] have been applied to a variety of fields, and related software and useful mathematical models have been developed [23,24]. In this paper, we will mainly review recent progress on development of QSPR for surfactants.

## 2. Studies on Relationships between CMC and Molecular Structures

In chemistry, the critical micelle concentration (cmc) is defined as the concentration of surfactants above which micelles are spontaneously formed. Upon addition of surfactants into a system, they will initially partition into the interface, reducing the system free energy by lowering the energy of the interface and by removing the hydrophobic parts of the surfactant from contacts with water. When the surface coverage by the surfactants increases and the surface free energy (surface tension) has decreased, the surfactants start aggregating into micelles, thus again decreasing the system free energy by decreasing the contact area of hydrophobic parts of the surfactant with water. Upon reaching cmc, any further addition of surfactants will just increase the number of micelles (in the ideal case). As a measurable physical quantity for sudden change in surfactant solution, cmc can be used as a gauge for the surface activity of surfactants. In the beginning of development of QSAR models for surfactants, the length of lipophilic chain of surfactants was mainly considered, e.g., Becher *et al*. [25] established an equation to relate cmc to the numbers of carbons and ethoxyl groups. In recent years, topological and electronic structures have been adopted for this purpose. The program CODESSA (Comprehensive Descriptors for Structural and Statistical Analysis), running under the Windows environment and developed by Florida University, can produce more than 400 molecular descriptors including constitutional, topological, geometrical and electronic structure, and this program can be combined with molecular orbital program MOPAC (Molecular Orbital PACkage) [26–29].

### 2.1. QSPR Studies on CMC of Surfactants Based on Molecular Connectivity Index

**Figure 1.**
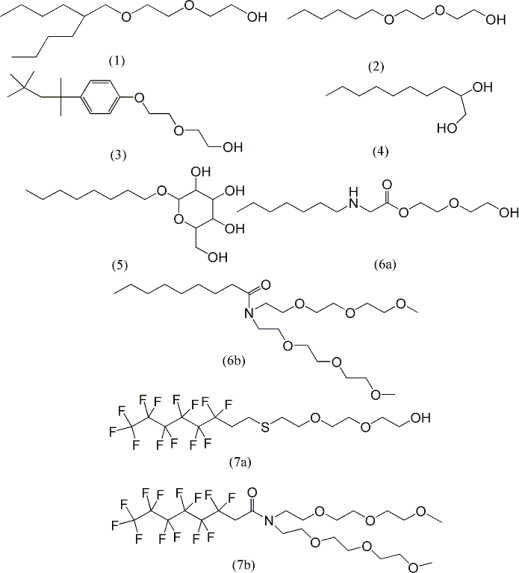
Representative structures of the seven nonionic surfactant classes used by Huibers *et al.* to predict the cmc for a series of 77 surfactants [30]: (1) branched alkyl ethoxylates (2) linear alkyl ethoxylates (3) octylphenol ethoxylates (4) alkanediols (5) alkyl mono and disaccharide ether and esters (6a) ethoxylated alkylamines (6b) ethoxylated alkylamides (7a) fluorinated linear alkylethoxylates (7b) fluorinated ethoxylated amides.

Using the CODESSA program, QSAR models have been developed by Huibers *et al*. to predict cmc values for non-ionic and anionic surfactants [30,31]. For non-ionic surfactants, a dataset of 77 samples was selected and divided into seven categories based on the characteristics of their hydrophobic and hydrophilic moieties [30] (Figure 1).

Multiple linear regression (MLR) analyses of molecular descriptors and the logarithm of the cmc were carried out using the heuristic algorithm, and the best model obtained is:

(1) $$\begin{array}{c} {\text{Log}}_{10}\text{cmc}=-(1.80\pm 0.16)-(0.567\pm 0.009)\text{c}-\text{KH}0+(1.054\pm 0.048)\text{c}-\text{AIC2}+(7.5\pm 1.0)\text{RNNO}\\ \left(n=77,\;R^{2}=0.9833,\;F=1433,\;S^{2}=0.0313\right) \end{array}$$

where n is the number of compounds used for regression, R^2^ the squared correlation coefficient, *S*^2^ the standard error of the regression, and F the Fisher ratio for the regression; c-KH0 stands for the Kier & Hall molecular connectivity index of zero-th order for hydrophobic fragment [32], and using the numbers of total electrons, valence electrons and hydrogen atoms contained in this fragment represents the contribution from all non-hydrogen groups; the second order average structural information index (c-AIC_2_) reflects basic chemical characteristics of hydrophobic moiety [33]; RNNO (so-called relative number of oxygen and nitrogen atoms) stands for the size of hydrophilic moiety and its value is related to the numbers of nitrogen and oxygen atoms. The positive regression constants (*R*^2^ = 0.9833) show that branches and other structures can increase cmc more than a straight chain.

**Figure 2.**
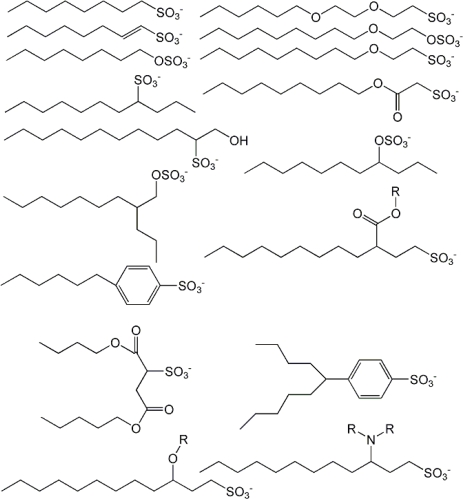
Representative structures of the anionic surfactants, showing the diversity of the hydrophilic and hydrophobic domains [30].

For the study of anionic surfactants, a dataset of 119 samples was selected, and molecular structural types under research are shown in Figure 2. The equation obtained is [31]:

(2) $$\begin{array}{l} {\text{Log}}_{10}\;\;\text{cmc}=(1.89\pm 0.11)-(0.314\pm 0.010)t-\text{sum}-{\text{KH}}_{0}-(0.034\pm 0.003)\text{TDIP}-(1.45\pm \\ 0.18)h-\text{sum}-\text{RNC}\\ \left(n=119,\;\;R^{2}=0.940,\;\;F=597,\;\;S^{2}=0.0472\right) \end{array}$$

In this equation, the most useful descriptor is t-sum-KH0 which represents the kier & hall index of zero-th order for the whole hydrophobic domain and is related to molecular volume and surface domain. The second descriptor TDIP is molecular total dipole moment obtained from atomic charges using MOPAC program [34]. Analysis of the change in total dipole moment of the anionic surfactants shows that total dipole moment increases with a rise in the size of alkyl chain if the hydrophilic moiety remains identical [30]. If the alkyl chain keeps unchanged, moving of hydrophilic group towards the molecular center will lead to a decrease of total dipole moment, however its effect on the cmc concentration is insignificant. For the double-tailed surfactants, total dipole moment is decided by the longer hydrophobic chain. The third descriptor stands for the sum of carbon atoms in the whole hydrophilic moiety and describes the variation of the hydrophilic moiety structure.

When the hydrophilic moiety is sulfate or sulfonate, change in cmc only depends on the hydrophobic moiety, and the change in the hydrophilic moiety is small and even a fragment descriptor is not needed for this. The regression equation is [30]:

(3) $$\begin{array}{c} {\text{Log}}_{10}\text{cmc}=(2.42\pm 0.07)-(0.537\pm 0.009)\text{KH}1-(0.019\pm 0.002){\text{KS}}_{3}+(0.096\pm 0.005)\text{HGP}\\ \left(n=68,\;R^{2}=0.998,\;F=1691,\;S^{2}=0.0068\right) \end{array}$$

In this model, the three descriptors are related to variation of the hydrophobic moiety of the surfactants. KH1 is the first-order Kier & Hall molecular connectivity index and is related to molecular surface domain and volume. KS3 is the third-order Kier & Hall molecular connectivity index, which contains the information for molecular shape. This index has a higher value for molecules with a straight chain than for those with a branched chain. HGP is hydrophobic group position on the longest chain, which simply describes the carbon number attached to the hydrophilic moiety. This descriptor explains the phenomenon that the cmc increases as the head group moves farther from the α-carbon position.

### 2.2. QSPR Studies of CMC of Surfactants Based on Quantum Mechanical Descriptors

It is known that dissolving of a surfactant in water and micelle formation is an exothermic process due to free energy reduction in the system [17], and that change in the energy mainly comes from interactions between surfactant molecules and between surfactant molecules and water molecules. Coulombic interactions, intermolecular van der Waals interactions and hydrogen bonding all play important roles in the formation of micelles. In the work by Wang *et al.* [35], therefore, quantum mechanical descriptors like molecular energies and dipole moment were additionally employed as descriptors and a better model was obtained between molecular structures and cmc. The model for the nonionic surfactants is [35]:

(4) $$\begin{array}{c} \text{lgcmc}=1.930-0.7846KH0-8.871\times 10^{-5}E_{T}+0.04938D\\ \left(n=39,\;R^{2}=0.9948,\;S^{2}=0.1232\right) \end{array}$$

The model for the anionic surfactants is [27]:

(5) $$\begin{array}{c} {\text{Lgcmc}}_{\text{cal}}=0.546-0.269KH0-0.00370\Delta H_{f}+0.000224E_{t}+0.382E_{HOMO}+0.493E_{LUMO}-0.0134D\\ \left(n=40,\;R^{2}-0.9778,\;F=74.72,\;S^{2}=0.1184\right) \end{array}$$

where E_T_ is the total energy of molecule, ΔH_f_ molar heat of formation, D the molecular dipole moment, E_HOMO_ the energy of the highest occupied molecular orbital, E_LUMO_ the energy of the lowest unoccupied molecular orbital, and KH0 the kier & hall molecular connectivity index of zero-th order. In the QSAR model for anionic surfactants, the molecular structure descriptors having effecs on the cmc are in the following order: KH0 > E_T_ > D > E_LUMO_ > ΔH_f_ > E_HOMO_. Katritzky *et al*. also suggested that significantly important molecular descriptors in the selected QSPR models were topological, solvational and charge-related descriptors as the driving force of the intermolecular interactions between anionic surfactants and water [36].

The correlation coefficients (R^2^) between the calculated values with the above two models and the experimental values are 0.9965 [35] and 0.9989 [27]. These high correlations have demonstrated the necessity of using electronic structures to study QSAR for surfactants.

Wang *et al*. [37] conducted a further study to derive a quantitative structure-property relationship for 77 nonionic surfactants belonging to eight series, and they suggested that the best model contained four quantum-chemical descriptors (ΔH, D, E_HOMO_ and E_LUMO_), and two constitutional descriptors (the molecular weight of surfactant (M) and the number of oxygen and nitrogen atoms (n_NO_)), and one topological descriptor (KH0). Wang *et al*. [38] used the model (Equation 5) to predict cmc of three AE_3_SO_3_ compounds, and their results indicated that the calculated values were in accordance with their observed values.

Li *et al*. optimized hydrophobic–hydrophilic segment geometries of 98 anionic surfactants at ab initio RHF/6-31G(d) level, and obtained a quantum chemical dataset including charge density, energies of molecular orbital and dipole moment. The anionic surfactants employed include sodium alkyl sulfates, sodium alkyl sulfonates, sodium alkyl benzenesulfonates, and potassium alkyl carboxylates with a wide variety of hydrophobic structures [29]. Based on one constitutional descriptor and two quantum chemical descriptors, a significant QSPR model for cmc of anionic surfactants was obtained by MLR technique. The model they have established is [29]:

(6) $$\begin{array}{l} {\text{log}}_{10}\text{cmc}(\text{cal})=(1.89\pm 0.0671)+(-0.0697\pm 0.00151)N_{\text{T}}+(-0.0323\pm 0.0015)\mu +(0.381\\ \pm 0.0305)Q_{\text{C}-\text{max}}\\ \left(n=98,\;\;R^{2}=0.980,\;\;F=1505.23,\;\;S^{2}=0.0107,\;\;R_{CV}^{2}=0.978\right) \end{array}$$

where *N*_T_ represents the total atom number in the hydrophobic–hydrophilic segment, μ is the dipole moment of surfactant segment, and *Q*_C-max_ represents the maximum net atomic charges on C atom; $R_{\mathrm{CV}}^{2}$ is the squared correlation coefficient for the ‘leave-one-out’ cross-validation procedure. Coefficient $R_{\mathrm{CV}}^{2}$ (0.978) indicates the excellent capability and stability of the regression equation developed. They concluded that the total atom number (*N*_T_) in the surfactant hydrophobic–hydrophilic segment plays a major role in the model, while the dipole moment (μ) of the surfactant segment and the maximum net atomic charge on C atom (Q_C-max_) in the surfactant segment are also important.

Katritzky *et al*. explored a data set of 181 diverse anionic surfactants to relate the logarithm of critical micelle concentration (cmc) to the molecular structure using CODESSA Pro software [36]. Their final regression equation involved five descriptors: the Kier & Hall index (order 1); the Kier shape index (order 2) defined for the hydrophobic fragment; moment of inertia B, calculated for the hydrophilic fragment; the total point-charge component of the molecular dipole; and the image of the Born solvation energy defined for the whole molecule. The most obvious influence on cmc was manifested by hydrophobic fragments expressed by the topological and geometrical descriptors, while the hydrophilic fragment is represented by constitutional, geometrical, and charge related descriptors.

### 2.3. QSPR Studies of CMC of Surfactants Using Neural Network

Utilizing MLR and an artificial neural network (ANN) algorithm, Katritzky *et al.* derived linear and nonlinear predictive models from a data set of 162 nonionic surfactants [19]. The artificial neural network (ANN) is a popular tool in function learning due to its ability to learn rather complicated functions. ANN is a mathematical model or computational model that tries to simulate the structure and/or functional aspects of biological neural networks. Neural networks are non-linear statistical data modeling tools. They can be used to model complex relationships between inputs and outputs or to find patterns in data. The descriptors in the derived models relate to the molecular shape and size and to the presence of heteroatoms participating in donor-acceptor and dipole-dipole interactions. Steric hindrance in the hydrophobic area also plays an important role in micellization. The QSAR models reported are expected to provide reliable estimations for the following surfactant classes: branched and linear alkyl ethoxylates, octylphenyl, ethoxylates, linear ethoxylated alcohols, octylphenols, alkanediols, alkyl mono- and disaccharides, ethoxylated alkylamines and alkylamides, fluorinated alkyl ethoxylates, carbohydrate derivatives, and dimeric surfactants.

A QSPR study was also performed using wavelet neural network (WNM) to relate the structure of 94 cationic Gemini surfactants to their cmc [16]. Wavelet neural networks are another novel approach towards the learning function.Wavelet networks, which combine the wavelet theory and feed forward neural networks, utilize wavelets as the basis function to construct the networks. The performance of the QSPR model obtained was investigated by the test set and the average error was 0.105 for the test set, which is superior to the MLR model. In this work, the cmc of Gemini surfactants was related to the 12 descriptors (seven topological, three WHIMs, one geometrical and one functional group descriptors) by WNN model for the first time.

### 2.4. QSPR Studies of CMC of Surfactants Using Other Methods

Considering the fact that relationship between free energy change in micellization process and the nucleus structure is related to hydrophobic fraction of surfactants, Robert *et al*. adopted octanol/water partition coefficient (logP) to systematically study the cmc models for anionic surfactants [39]. They made regression analysis of experimental cmc values for primary alcohol sulfate and primary alcohol ester sulfate (at 50 °C) vs IIh (the logP fragment value for the hydrophobe, simply defined as the whole molecule minus the negatively charged fundamental fragment SO_3_^−^ or OSO_3_^−^) and L (the length of the hydrophobe, in C-C single bond unit), and the following QSPR models were obtained [39]:

(7) $$\begin{array}{c} \text{Pcmc}=0.32(\pm 0.13){Л}_{\text{h}}+0.08(\pm 0.05)L-1.02(\pm 0.71)\\ \left(n=16,\;R^{2}=0.927,\;F=83,\;S=0.17\right) \end{array}$$

(8) $$\begin{array}{c} \text{Pcmc}=0.39(\pm 0.05){Л}_{\text{H}}+0.08(\pm 0.02)L-1.50(\pm 0.30)\\ \left(n=16,\;R^{2}=0.989,\;F=582,\;S=0.07\right) \end{array}$$

where Pcmc is the negative logarithm of the cmc. IIh in Model 6 was calculated with Leo and Hantsch approach [40] and IIh in Model 7 computed based on position-dependent breaching factor [41]. A regression was made between the cmc values calculated with Model 7 and the experimental cmc values, and a fairly good liner relationship was found. The correlations obtained are summarized as follows: for all anionic surfactants (n = 133, R^2^ = 0.976, S = 0.12, F = 5360); all anionic surfactants except SALS (secondary alcohol sulfates), LAS (linear alkyl benzene sulfonate), and β-branched PAS (primary alcohol sulfate) (n = 75, R2 = 0.988, S = 0.09, F = 6122); SALS, LAS and β-branched PAS (n = 58, R^2^ = 0.982, S = 0.08, F = 3074). In addition, molecular mechanics has also been used to predict cmc for surfactants like linear alkyl polyoxyethylene ethers and alkyl polyglucoside [42,43].

## 3. Charge Distribution of Surfactants and Its Influence on Their Properties

A variety of properties for surfactants are related to charge distribution in their molecules. A recent study showed that in colloidal dispersion systems (mainly system with low particle concentrations and high surface charges) there existed a long-range force purely from electrostatic interactions between particles in addition to short-ranged forces caused by van de Waals interaction [44]. Therefore the key to the study of electrostatic interactions is description and quantification of the charge distribution.

### 3.1. Computation of Charge Distribution in Ionic Surfactant Molecules and Their Effects

**Figure 3.**
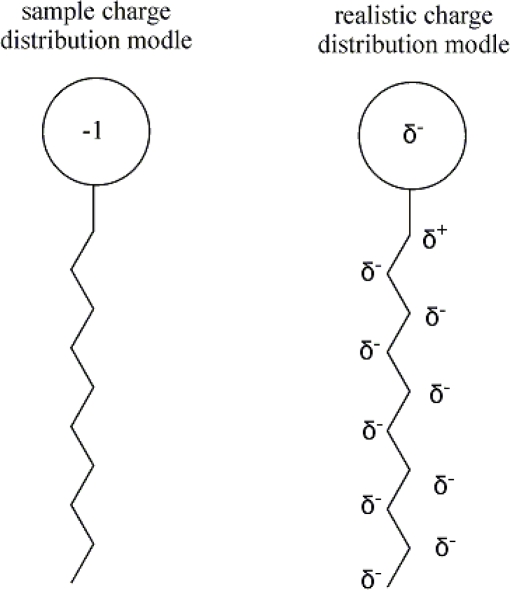
For ionic surfactants, quantum chemical calculations suggest that the charge is distributed throughout the molecule [45].

For ionic surfactants, net charge carried on the headgroup of the molecules has been normally treated as a point charge, although it is factually located on several atoms in the hydrophilic groups and even enter tails of surfactant molecules (see Figure 3) [45].

Using an *ab initio* 6-31G basis set, electrostatic potential surface was calculated for dodecyl carboxylate and decyl sulfate and proved that there is a partial negative charge on the alkyl chain of these surfactants [46]. By studying the fluorescence quenching behavior and constants of ionic surfactants, it is indicated that the single molecule of the surfactant adopt dynamic coil configuration in water [47]. This configuration will be more closely related to molecular charge distribution than other configurations. Consequently, a previous assumption that the tail part of a surfactant is nonpolar and electronically in equilibrium is not objective in a physical sense.

**Figure 4.**
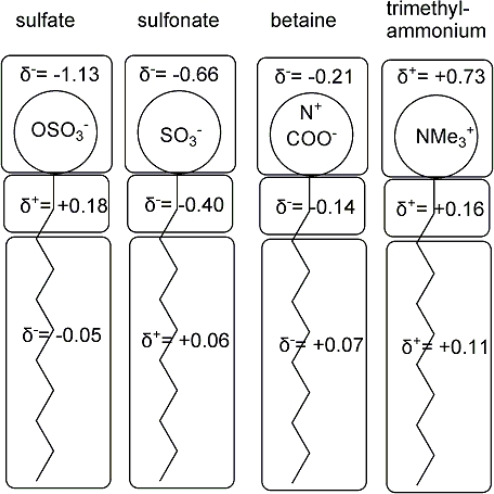
Distribution of charge in selected surfactants between the head group, the α-methylene, and the remaining portion [45].

Huibers has recently studied charge distribution in common ionic surfactants using four widely accepted semiempirical methods (MINDO/3, AM1, PM3 and MNDO/d), and developed QSAR models for some properties of these surfactants (Figure 4) [45]. The ionic surfactants they studied include anionic (sulfate, sulphonate and carboxylate), cationic (trimehthylammonium and pyridinium) and amphoteric (betaine and dimethylamine oxide) classes. Addition of d-orbital to basis sets for MNDO [48] has improved calculation results for elements of the third period such as sulphur and phosphorus. From the calculation results, it can be seen that there is a partial charge distribution found for the α-CH_2_ and the alkyl chain. The terminal methyl group of all surfactants has a positive charge and this causes their neighboring CH_2_ to carry a partial negative charge. For the anionic surfactants, a ca. 5% partial negative charge resides on the tail part of the molecules (for sulphonate surfactants, a 5% partial positive charge is carried on the tail part of the molecules). The polar head of the amphoteric surfactants carries a ca. 6% negative charge with the same amount of positive charge on the tail. The polar head of the cationic surfactants carries a positive charge and the tail part of surfactants also carries a ca. 10% positive charge, the highest local charge. Although, in some studies modeling associated behavior of surfactants [49], α-CH_2_ was considered as part of the polar head, the explanation about this was not given.

NMR data from Zhao and Fung [50] also indicated that chemical environment around the α-CH_2_ is different from those around the other methylene groups and this may lead to its association with water molecules. Huibers *et al.* have quantified charges on these groups and shown that a relatively high amount of charge is located on the α-CH_2_ and thus have supported the idea that the α-CH_2_ belongs to the polar head group [45]. In addition, it is interesting to note that the sign of the charge on the α-CH_2_ and that of the charge on polar head can be the same or opposite. These results showed that existence of local charge on the tail of the alkyl chain of surfactants provide a need to reconsider the properties for micelle cores. Normally, for treatment of micelle formation and solubilization, ‘water drop’ model has been employed without consideration of repulsive interaction between the alkyl chains [49,50]. Obviously, the migration of the charge on the polar head of ionic surfactants to the other part of the molecules, especially to α-CH_2_ and terminal methyl group, has an important effect on their properties. Polar head charge and charge on α-CH_2_ have varying effects on cmc of different surfactants. Inequality of local charges on the alkyl chains can rationalize polarity of micelles and effects of polar head charge on molecular self-assembly.

Furthermore, using the Huckel molecular orbital theory, Jacobs and Anacker have computed charges on hydrophilic group for decyl pyridinium chloride [51], finding that the aggregation numbers in micelles is related to atomic charges on the pyridinium ring attached to the alkyl chains. They were trying to elucidate the role of charge delocalization in micelle formation by determining the aggregation numbers of 1-decylpyridinium bromide and three of its structurally similar isomers (2-, 3-, and 4-decylpyridinium hydrobromide) in an aqueous environment, and suggested that the positive charge is not localized on the nitrogen but is spread over the entire polar head. Using the AM1-calculated charges, Huibers and Jacobs [52] have rationalized the effect of the charge distribution on the hydrophilic group on the aggregation numbers in micelles. The aggregation numbers of the surfactants are shown to increase with a decrease in the residual partial charge in the alkyl tails, suggesting a change in the packing of the surfactants. The critical micelle concentration increases with a decrease in the partial charge of the head groups, indicating increased solubility of the surfactant molecule as charge is more widely distributed throughout the molecule. Villamagna *et al.* [53] have studied configuration design for structure of surfactants for water-in-oil emulsions using AM1 calculations. Their molecular modelling analysis of presently used surfacts in water/oil emulsions leads to the ideal structure of a surfactant have tail:polar head:hydrogen bonding chains in the volume ratio 1:1:1, and that is a useful way to designing ideal surfactants.

In the meantime, it should be realized that the above calculations are based on gaseous-phase models. Although no environmental effects are considered in the calculations and only pure charge distribution is provided, this can be used to compare different surfactants. To consider the environment’s perturbation on charge distribution, *i.e.*, to model the solvation environment is a fairly complex process, since this kind of models needs to reflect the characteristics of flowing media with a certain dielectric strength. Consequently, further studies on model establishment theory and calculation capability are needed in order to predict such a complex system as surfactant micelles.

### 3.2. Descriptors Related to Molecular Surface Area—CPSA

To study interactions between molecules, some researchers have used solvent-accessible molecular surface area as a descriptor. There have been some reports on descriptors for molecular surface area, atomic charges and charge scaling factors and their applications [54–56]. Stanton and Jurs [57] have combined molecular surface area with atomic charges and defined a new molecular descriptor named charged partial surface area (CPSA) to address polarization interactions between molecules, which can be correlated with physical quantities such as chromatographic retention, boiling point and surface tension.

The geometric model for CPSA descriptor is to utilize overlapping of hard spheres defined by the van de Waals radii of atoms. The calculation for this descriptor was performed with a UNIX system (Sun4/1102) and ADAPT software. Solvent-accessible area calculation adopted SAVOL algorithm developed by Pearlman [58]. Atomic charges were obtained from the Abraham and Smith [59] algorithm, an empirical method including σ and π contributions, which is parameterized to reproduce experimental dipole moments. Calculations of both surface areas and atomic charges include hydrogen atoms. The CPSA descriptor system established in this study have 25 individual descriptors, which include partial positive surface area descriptors (PPSAs), partial negative surface area descriptors (PNSAs), partial surface area descriptors (DPSAs), fractional charged surface area descriptors (FPSA and FNSA), total surface weighted partial surface area descriptors (WPSA and WNSA), relative positive and relative negative charges descriptors (RPCG and RNCG), and relative positive and relative negative charged surface area descriptors (RPCS and RNCS).

## 4. Surface Tension Prediction Models

Surfactants can to some extent balance interfacial unsaturated force fields to reduce surface tension. Different structures of surfactants and the resulted difference in intermolecular interactions can be understood at different levels. The Wang group has focused on parameters of molecular structure of surfactants [60], used as molecular descriptors oxygen atom number (NO) in hydrophilic group, Kier & Hall zeroeth-order index (KH0), heat of formation (ΔH_f)_ calculated by quantum mechanics, total energy of a molecule (E_T_), molecular mass (Ws) and dipole moment (D) etc to regress *vs.* minimum surface tension at cmc (γ^0^) and established several types of related models. Among them, the best model is [61]:

(9) $$\begin{array}{c} \gamma^{0}=11.98+0.4780N0+0.5848KH0-0.0007763E_{T}-0.01053\Delta {\text{H}}_{\text{f}}+0.09734\text{D}-0.1345N0.\;KH0\\ \left(n=30,\;R^{2}=0.9945,\;F=187.8,\;S^{2}=0.5302\right) \end{array}$$

Their chosen data set of γ^0^ contains 30 diverse structures of nonionic surfactants and molecular descriptors ΔH_f_, E_T_ and D are obtained from calculations using MNDO-MOPAC 7.0. Through energy and electronic parameters, this model set up indirect QSAR between surface tension and electron motion.

Wang *et al*. have also established quantitative models for 20 anionic surfactants with different structures concerning their surface tension reduction effect expressed as surface pressure (II) at different temperatures (t) and different counter ion concentrations (c) [62]. This effect is normally expressed as maximum surface pressure measured at cmc condition. In addition to temperature and counter ion concentration, variables for the optimal models obtained also include molar heat of formation for anionic surfactant (ΔH_f_), dipole moment (D) and Kier & Hall zeroeth-order index (KH0) for hydrophobic moiety. The model containing five descriptors is as follows [63]:

(10) $$\begin{array}{c} \text{IIcmc}=27.71-0.005239\Delta H_{\text{f}}+17.16\text{C}-0.1520\text{T}-0.2130\text{D}+1.080\text{KH}0\\ \left(n=20,\;R^{2}=0.9884,\;F=56.43,\;S^{2}=0.0134\right) \end{array}$$

The IIcmc_cal_ values calculated with this model is highly related to the observed values, and the regression equation is as follows:

(11) $$\begin{array}{c} {\text{IIcmc}}_{\text{cal}}=2.982+0.9199{\text{IIcmc}}_{\left(\text{obs}\right)}\\ \;\;\;\;(n=34,\;\;R=0.994,\;\;S^{2}=0.0688) \end{array}$$

Stanton *et al.* [64] and Stanton and Jurs [65] have focused on descriptors relating to molecular surface area. They made multiple linear regressions on observed surface tension of alkanes, alkyl esters, alkyl alcohol, *etc.* against these descriptors, and established surface tension prediction models. 146 compounds were selected for the regressions, among which 74 compounds are from alkane type (accounting for 50.7% of the total), 44 compounds from α-ester type (accounting for 30.1%), and 28 compounds from alcohol type (accounting for 19.2%) [64]. This model contained 10 molecular descriptors [65], among which six descriptors are topological, two electronic and two from hydrogen bonding system. Predicted surface tension values for these three systems under study showed an excellent correlation with the experimental values (R^2^ = 0.983, s = 0.4 dyn/cm). After studying inter-correlations of these descriptors, these authors indicated that the topological descriptors are of a significant correlation to the molecular surface area, and intermolecular interaction increases with a rise in the molecular surface area. This will result in a relatively high surface tension, and thus molecular surface area is the most influential factor for surface tension.

## 5. Qsar Studies on Cloud Point of Nonionic Surfactants

The cloud point of a nonionic surfactant is the temperature where the mixture starts to phase separate and two phases appear, thus becoming cloudy. This behavior is characteristics of non-ionic surfactants containing polyoxyethylene chains, which exhibit reverse solubility *versus* temperature behavior in water and therefore “cloud out” at some point as the temperature is raised. It is affected by salinity, being generally lower in more saline fluids. Cloud point is a critical factor in the performance of nonionic surfactants (such as those containing polyoxyethylene polymers as their hydrophilic moieties) in detergent formulations [66,67]. Nonionic surfactants show rich phase behavior in aqueous mixtures. Below their cloud points (CPs), a number of isotropic phases exist. Above their CPs, nonionic surfactants form opaque suspensions, which eventually separate into water-rich and surfactant-rich phases [68,69].

Bünz *et al.* studied cloud point of 20 nonionic surfactants with alkyl zwitterions groups, obtaining QSAR models (Table 1) [70]. These models contained four descriptors for molecular structures: two topological [average information content (order 2), Kier shape index (order 3)] and two constitutional descriptors (relative molecular weight, relative number of rings).

The squared correlation coefficient for this model was R^2^ = 0.9948. Huibers *et al*. have established an empirical relationship to estimate the cloud point of pure nonionic surfactants of the alkyl ethoxy with 41 topological descriptors, and the best regression was [71]:

(12) $$\begin{array}{c} CP=(-264\pm 17)+(86.1\pm 3.0)\text{log}\text{EO}\#+{\left(8.02+0.78\right)}^{3}k-(1284\pm 86{)}^{0}\text{ABIC}-(14.26\pm 0.73{)}^{1}\text{SIC}\\ \left(n=62,\;\;R^{2}=0.937,\;\;F=211,\;\;{\text{S}}^{2}=42.3\right) \end{array}$$

where EO# is the number of ethylene oxide residues, ^3^k is the third order Kier shape index for the hydrophobic tail, ^0^ABIC is the zeroth order average bonding information content of the tail, and ^1^SIC is the first order structural information content of the tail.

In 2003, Yuan *et al*. developed another equation to predict cloud point for nonionic surfactants with their several structural, electronic, spatial and thermodynamic properties, and their best regression was [72]:

(13) $$\begin{array}{c} T_{c}=9.62958+0.69733A+0.981001\mu -y-1.59247\mu -z-19.0815\;\;\text{lg}P-0.829297M_{\text{r}}\\ \left(n=49,\;\;R^{2}=0.844,\;\;F=46.618\right) \end{array}$$

where lg*P* is the octanol/water partition coefficient, A is the molecular area, *M*_r_ is the relative mass, and *μ-y* and *μ-z* were the molecular dipole.

In 2006, Ren *et al.* developed the QSPR models to predict cloud points and study the cloud phenomena of nonionic surfactants in aqueous solution [69]. Four descriptors were selected by the heuristic method as the inputs of multiplier linear regression and support vector machine (SVM) models. The basic idea of SVM is to map the input vectors into a higher dimensional feature space by a kernel function, *K(xi, xj)*, and then to do linear regression in this space. SVM models performed better both in fitness and in prediction capacity. For the test set, they gave a predictive correlation coefficient of 0.9882, root mean squared error of 4.2727, and absolute average relative deviation of 9.5490, respectively. The proposed models can provide some insight into what structural features are related to the cloud points of compounds, *i.e.*, the molecular size, structure, and isomerism of the hydrocarbon moiety and the degree of oxyethylation.

More recently, QSPR analysis has been directed to a series of pure nonionic surfactants containing linear alkyl, cyclic alkyl and alkyl phenyl ethoxylates [66]. Modeling of cloud point of these compounds as a function of the theoretically derived descriptors was established by MLR and partial least squares (PLS) regression. PLS, which is based on factor analysis fundamentals, is applied where there are many variables but not enough samples or observations. PLS has been applied to many fields of applied sciences with great success. In chemometrics, it is one of the favored methods of analysis. In this study, a genetic algorithm (GA) was employed as a variable selection method in QSPR analysis. GA is developed to mimic some of the processes observed in natural evolution, which are an efficient strategy to search for the global optima of solutions. The results indicate that the GA is a very effective variable selection approach for QSPR analysis. The comparison of the two regression methods used showed that PLS has better prediction ability than MLR.

## 6. Studies on Degradation of Surfactants

QSPR studies with respect to cmc, charge distribution and surface tension prediction of surfactants have received a high attention as described above. However biodegradation potential of surfactants should be another highly important issue as this decides their environmental impact. Concerning biodegradation of surfactants, the reported studies include effects of alkyl chain structure (straight and branched chains) and position of branching in the chain, *etc.*, on biodegradable activity [73–75]. Biodegradation is the process whereby organic (*i.e.*, carbon-containing) matter is decomposed by the action of micro-organisms present in the environment. The evaluation of biodegradability of anthropogenic organic substances is an essential parameter for environmental risk assessment and required according to appropriate legislation.

Biodegradation with respect to surfactants is defined as primary biodegradation, ultimate biodegradation and ready aerobic biodegradability. Primary biodegradation means the structural change (transformation) of a surfactant by microorganisms resulting in the loss of its surface-active properties due to the degradation of the parent substance. Ultimate biodegradation means the level of biodegradation achieved when the surfactant is completely used by micro-organisms resulting in its breakdown to inorganic end-products such as carbon dioxide, water and mineral salts of any other elements present (mineralization) and new microbial cellular constituents (biomass). Ready aerobic biodegradability is an arbitrary classification of surfactants which have passed certain specified screening tests for ultimate biodegradability; these tests are so stringent that it is assumed that such [76,77].

Siwiski *et al*. developed a modified river water die-away test for controlling the biodegradability of anionic surfactants and non-ionic surfactants of detergent powders and investigated twelve powders. They found that anionic surfactants were much more easily biodegraded than non-ionic surfactants, and non-ionic surfactants were very different in terms of biodegradability [78]. Sales *et al*. have carried out a study to research the influence of several environmental factors on the biodegradation of a commercial anionic surfactant (LAS) in waters and sediments of Cadiz Bay (southwest Iberian Peninsula). They concluded that degradation is basically an aerobic process, and hence the introduction of air to the solution will favour it [79]. Li *et al*. have conducted a study to interpret the differences in biodegradation of LAS and its coproducts from the electronic structure characteristics and to explore the mechanism of LAS biodegradation. In their research, electronic descriptors of LAS and its cocproducts, including orbital energy, dipole moment, charge distributions and local electronic characteristics of surfactant molecule were calculated by using semiempirical quantum chemical method at the PM3 level. They have explained why biodegradation of model compounds at first takes place in the terminal CH_3_ group, and they have given two reasonable explanation: (1) there is no transferred H atom in the S-O bond; (2) the degrees of S-O bonding are higher than those of the terminal C-H bonding in the model compounds [80].

PFC surfactants can resist degradation by acids, bases, oxidants, reductants, photolytic processes, microbes and metabolic processes [81–84]. Some monitoring studies indicate that fluorosurfactants are globally distributed, environmentally persistent and bioaccumulative [85,86]. To evaluate the fate of PFCs in the environment a set of principal transformations was developed and implemented in the simulator of microbial degradation using the catabolite software engine (CATABOL) [87]. The simulator was applied to generate metabolic pathways for 171 perfluorinated substances on Canada's domestic substances list. It was found that although the extent of biodegradation of parent compounds could reach 60%, persistent metabolites could be formed in significant quantities. During the microbial degradation a trend was observed where PFCs are transformed to more bioaccumulative and more toxic products. Perfluorooctanoic acid and perfluorooctanesulfonate were predicted to be the persistent biodegradation products of 17 and 27% of the perfluorinated sulphonic acid and carboxylic acid containing compounds, respectively.

Structural, electronic, and thermodynamic properties of linear perfluorooctane sulfonate (PFOS) and its trifluoromethyl-branched isomers (*i.e.*, 1-CF_3_– to 6-CF_3_–PFOS) were theoretically investigated by density functional theory (DFT) calculations with the B3LYP functional and a 6-31++G(d,p) basis set [88]. The linear and branched PFOS ions were identified as the most suitable compounds for interacting with charged species. Furthermore, in the linear anion, the LUMO orbital is located along the whole fluoro-carbon chain, while it is localized to the region close to the ternary carbon in the 4-CF_3_–PFOS, 5-CF_3_–PFOS, and 6-CF_3_–PFOS isomers.

The higher accessibility of the LUMO orbital in these branched anions implicates that they have a higher probability of reacting with free radicals compared with the linear counterpart. This finding is in agreement with the experimental observation that only the branched PFOS isomers were susceptible to reductive defluorination by reduced vitamin B_12_ as previously reported. The relative stability of the linear and branched PFOS in their different forms computed by comparing their calculated Gibbs free energy showed that 1-CF_3_–, 6-CF_3_–, and linear PFOS are the most favorable structures in terms of chemical stability.

In summary, QSPR studies on degradation of surfactants are far from complete, and further studies should be initiated especially on fluorosilicone surfactants and silicone surfactants. Due to their higher degradation potential than those of PFOS and PFOA, they belong to a promising direction, where QSPR method can be actively applied for studying their degradation-related properties.

## 7. QSPR Studies on Other Properties of Surfactants

It is well known that surfactants are typically amphiphilic molecules that contain both hydrophilic and lipophilic groups. The hydrophile-lipophile balance (HLB) is one of the indicators representing the ratio of the hydrophilicity of a surfactant to its hydrophobicity. The value of HLB number is between 0–60 defining the affinity of a surfactant for water or oil. Chen *et al.* have established two QSPR models for the HLB value of anionic surfactants by using the quantum chemical descriptors generated by semiempirical approach and density functional theory (DFT). One multiple linear regression model 14 included 46 anionic surfactants belonging to four series of alkyl sulfates and alkyl sulfonates, with the optimal squared correlation coefficient (R^2^) being 1.000, and the other multiple linear regression model 15 involved 73 structures including polyoxyethylene, acetate, propionate and fluorinated anionic surfactants, with the optimal squared correlation coefficient being 0.993 [89].

(14) $$\begin{array}{c} {\text{HLB}}_{\mathrm{cal}.}=-59.789+27.223N_{\text{O}}+0.00898E-0.0424D-38.474E_{\text{HOMO}}\\ \left(n=46,\;\;R^{2}=1.000,\;\;F=130259.2,\;\;S^{2}=0.0027\right) \end{array}$$

(15) $$\begin{array}{c} {\text{HLB}}_{\mathrm{cal}.}=552.760+205.512N_{\text{O}}-664.509N_{\text{O}}^{1/2}-0.101D+47.809E_{\text{HOMO}}+0.203D-y\\ \left(n=73,\;\;R^{2}=0.993,\;\;F=1780.713,\;\;S^{2}=0.015\right) \end{array}$$

It is noteworthy that using these quantum mechanical descriptors can differentiate the differences between the HLB values of different isomers of the surfactants and overcome the difficulty encountered by Davies equation (HLB = ∑(hydrophilic group numbers) + ∑(hydrophobic group numbers)). Davies in the 1950s developed a system based on the analysis of group numbers. The “group number” characterizes the contribution of each specific functional group to the energy that would be required if a solvent molecule were changed from water to an organic solvent [90].

Liu *et al*. have established a QSPR model between molecular electronegativity—distance vector (MEDV) and HLB Values of anionic surfactants. Their model was examined by both internal and external validation on its stability, and the details were shown in Equation 16 [91]:

(16) $$\begin{array}{l} \text{HLB}=52.775+18.185M_{11}-2.423M_{12}-9.151M_{13}-0.333M_{22}-22.746M_{24}-246.157M_{33}\\ -41.806M_{34}-48.796M_{44}\\ \left(n=65,\;\;R=0.970,\;\;F=112.886,\;\;SD=3.232,\;\;Rcv=0.957,\;\;F\text{cv}=75.540,\;\;SD_{\text{cv}}=3.895\right) \end{array}$$

Ghasem *et al*. established some QSPR models to predict solubility of nonionic solutes in anionic micelle, and their QSPR models were tested for an external prediction set of 11 compounds randomly chosen from 62 compounds. The squared regression coefficients of prediction for the multiple linear regression and partial least squares regression methods were 0.9679 and 0.9728 respectively [92].

Campbell *et al.* studied effects of surfactants on attachment of bacteria to cellulose acetate (CA) and aromatic polyamide (PA) reverse osmosis membrane [93]. They analyzed effects of 23 classes of surfactants, including nonionic, anionic and amphoteric types, on attachment of Mycobacterium Sp to CA and PA membrane. The results showed that 17 classes of surfactants inhibited attachment of the bacteria to PA membrane, 25 classes inhibited the attachment to CA membrane, and 13 classes inhibited the attachment to both CA and PA membrane. Results from examination of adsorption of anionic surfactants to CA membrane using ATR-FT/IR (attenuated total reflection fourier-transform infrared) indicated that structures of surfactants can be effectively manipulated to optimize adsorption to inhibit attachment of the bacteria to reverse osmosis membrane. Also using SciQSAR program (SciVision, Lexington, MA) to calculate structures of surfactant molecules, 17 molecular descriptors were obtained ranging from molecular weight to electronic and topological ones. The QSAR models obtained for attachment force of surfactants to CA and PA membrane is [93]:

(17) $$\begin{array}{l} \text{CA}\;\text{membrane}:\;\text{attachment}\;\text{force}=1.06\times 10^{-3}+1.01\times 10^{-5}\text{CMC}+4.52\times 10^{-5}\;\text{Dipole}+\\ 4.35\times 10^{-4}{\text{ABSQ}}_{\text{on}}+0.0187\text{MaxQpos}-8.00\times 10^{-5}{\text{K}}_{\text{a}}3\\ \left(n=23,\;\;R^{2}=0.377\right) \end{array}$$

(18) $$\begin{array}{c} \text{PA}\;\text{membrane}:\;\text{attachment}\;\text{force}=0.13-6.40\times 10^{-4}\text{CMC}-0.0407\text{log}\text{P}-1.89\times 10^{-3}\;\;\text{Dipole}\\ \left(n=23,\;\;R^{2}=0.771\right) \end{array}$$

where ABSQ_on_, M_ax_Q_pos_ and K_a_3 stand for the sum of absolute values of charges on nitrogen and oxygen atoms in a surfactant molecule, the largest charge on all atoms and the third-order Hall & Kier index, respectively. Sensitivity analysis of the variables above demonstrated that for CA membrane system, K_a_3 and cmc were the most effective factors to determine surface active effects, while dipole moment and other descriptors had very limited effects. For PA membrane system, cmc was a strongest factor, and dipole moment and lgP had very limited effects.

Structure of alkyl chain, especially the chain length, has a direct or indirect effect on properties of a surfactant. By using the methods of quasielastic light scattering spectroscopy, Biz and Occelli derived hydrodynamic radius of alkyl sulphate micelle (Rh) [94]. On the basis of this, Missel *et al.* performed a theoretical study on the formation of rod-shaped micelles from sphere-shaped micelles [95], deriving the kinetic constant (K) for controlling dodecyl sodium sulfate micelle growth. Their further study manifested that K could work as function of chain length (the number of carbon atoms n_c_ = 8–12), R_h_ value increased linearly approximately and was more dependent on temperature with the chain length. At high concentrations of NaCl, the growth of micelles depends strongly on temperature, and when concentration of a surfactant is higher than cmc, micelles would be transformed into a cylindrical shape from a spherical shape by bonding to each other [96].

By changing surfactant concentration, washing temperature and washing time, Lindgren *et al.* studied correlation of detergency of some nonionic surfactants with these physicochemical properties and established a QSPR model to predict cleaning effect (Y) [97]:

(19) $${\text{Y}}_{\text{obs}}={\text{b}}_{0}+{\text{b}}_{1}\text{C}+{\text{b}}_{2}\text{t}+{\text{b}}_{3}\text{T}+{\text{b}}_{11}{\text{C}}^{2}+{\text{b}}_{22}{\text{t}}^{2}+{\text{b}}_{33}{\text{T}}^{2}+{\text{b}}_{12}\text{ct}+{\text{b}}_{13}\text{cT}+{\text{b}}_{23}\text{tT}+\text{e}$$

where c1, t, T stands for concentration of a surfactant, washing time and temperature, respectively. Coefficient b_0_ is constant; b_1_, b_2_ and b_3_ stand for contribution to cleaning effect from each respective variable; b_11_, b_22_ and b_33_ reveal whether in this effect, variable can give maximum/minimum values; b_12_, b_13_ and b_23_ stand for interaction of different variables. According to this model, detergency effect of surfactants is influenced mainly by (in order of importance): longest carbon chain in the hydrophobic part (redc); critical packing parameter where the branching of the hydrophobic part is taken into account (redcpp); hydrophilic-lipophilic balance (HLB); derivative of the cloud point curve (dCP); relationship between the longest carbon chain and the total amount of carbon in the hydrophobic part (redc/c) and amount of nonethoxylated fatty alcohol (f-alcohol). It was also shown that detergency effect of nonionic surfactants was related to cmc, molecular weight, unit of ethyleneoxy in the hydrophilic part and the number of carbon atoms in the hydrophobic part. Washing temperature was affected by the number of different carbon chains present in the hydrophobe (chains), cloud point, *etc.* These all indicated that detergency effect of surfactants was affected by molecular structures.

Warszynski and Lunkenheimer analyzed experimental results on surface tension for dimethyloxy phosphine with 7–13 carbon atoms in alkyl chain and its homologues [98], indicating that there was a repulsive interaction in adsorption layer and demonstrating that this was the result of decrease in configurational free energy due to a closer coiling of the hydrophobic chain. The adsorption isotherm for surfactants on air/water interface (statistical level) presented by these authors explicitly considered configurational free energy and the results exhibited that under a constant surface pressure, conformational free energy increased linearly with the hydrocarbon chain length.

Wang *et al.* investigated the interaction of CH_3_ (CH_2_)_7_OSO_3_^−^ with 1 to 6 water molecules at the air-water interface with quantum mechanics [99]. DFT (density functional theory) was employed to optimize the configuration of the anionic surfactant complexes CH_3_(CH_2_)_7_OSO_3_^−^ (H_2_O)*n* (*n* = 0–6) and calculate their molecular frequencies at the B3LYP/6–311+G* level. The results revealed that the hydration shell was formed in the form of H-bond between the hydrophilic group of CH_3_(CH_2_)_7_OSO_3_^−^ and 6 waters. The strength of H-bonds belongs to medium. Binding free energy revealed that the hydration shell was stable. The increase of the number of water molecules will cause increases of the total charge of hydrophilic group and S10-O9-C8 bond angle, but decreases of the alkyl chain length and the bond lengths of S10-O11, S10-O12 as well as S10-O13, respectively.

Based on linear solvation energy relationships (LSERs), Vitha and Carr studied fundamental chemical interactions responsible for solute retention in micellar electrokinetic capillary chromatography (MEKC) [100]. The system under study was homologous series of sodium dodecyl sulfate (SDS), sodium decyl sulfate (SdecS) and sodium octyl sulfate (SOS). It was found in this study that there was no evident change on interaction of solute with micelles when the number of carbon atom in the alkyl chain changed from 8–12. In fact, chromatographic parameters for the micelle phase of the three systems under study were identical. Analysis of linear solvation energy relationship and free energy change for each methylene transferring from water to micelles (ΔG^θ^_CH2_) indicated that steadiness of solvation free energy as function of alkyl chain length made SDS, SdecS and SOS have similar solvation energy. An important conclusion was drawn from this that the solute existed in polar hydrophobic moiety of micelles, rather than in the nonpolar core part. In addition, an effect of solute functional group on its internal positioning and orientation was also discussed in this work.

For predicting the interaction parameters $\delta_{t}^{2}$ of surfactants or organic substances in aqueous solution, by using MNDO-MOPAC7.0 software Wang *et al*. have obtained a quantitative structure-property relationship (QSPR) for 30 compounds belonging to six classes with their molecular forms as follows: C_n_H_2n+1_COOH, C_n_H_2n+1_OH, C_n_H_2n+1_NO_2_, CmH_2m+1_COC_n_H_2n+1_, n-C_m_H_2m+2_ and C_n_H_2n+1_ pyrrolidone [21]. By combining the principal component analysis (PCA) with the best multilinear regression analysis together in the heuristic method, multiple linear regression analysis among the more than 30 descriptors and $\delta_{t}^{2}$ was made. Principal component analysis (PCA) involves a mathematical procedure that transforms a number of possibly correlated variables into a smaller number of uncorrelated variables called principal components. The best correlation model contains 7 descriptors shown in Equation 20 [21]:

(20) $$\begin{array}{l} \delta_{\mathrm{tcal}}^{2}=58.274+0.009987\Delta H_{\text{f}}-0.01241E_{t}+0.0022905E_{\text{e}}+5.401E_{\text{HOMO}}+3.101E_{\text{LUMO}}+\\ 1.752KH0+576.2RNNO\\ \left(n=30,\;\;R^{2}=0.925,\;\;F=38.68,\;\;{\text{s}}^{2}=0.000\right) \end{array}$$

where *ΔH*_f_ is the heat of formation of molecule, *E_t_* is the total electronic energy, *E*_LUMO_ and *E*_HOMO_ are the energies of the lowest unoccupied molecular orbit and the highest occupied molecular orbit, *KH0* is the Kier and Hall index of zero order of the hydrophobic fragment of compound and *RNNO* is the relative number of oxygen and nitrogen atoms of the hydrophilic segment. *R*^2^, *F*, *s*^2^, and n are the correlation coefficient, the F-test, the standard error, and the number of the regression model, respectively.

It is known that when more than two surfactants with different molecular structures are mixed, their surface activities often can be increased intensively (synergism) [101]. The interaction between two surfactants is mainly due to electrostatic forces. The strength of attractive electrostatic interaction decreases in the order anionic–cationic > anionic–zwitterionic capable of accepting a proton > cation–zwitterionic capable of losing a proton > anionic–POE nonionic > cationic–POE nonionic. Mixtures of surfactants of the same charge type can show significant interaction at other interfaces interaction, although they show very weak effect at the aqueous solution–air interface [102]. Some studies addressed on this phenomenon have also been conducted. Tu *et al.* established two kinds of equations of the surface tension *vs.* the concentration for ideal binary mixtures of surfactants with Newton iterative method [103]. Their accuracy was verified by comparison among the values obtained respectively from the iterative, the numerical and observable approaches for surface tension of the ideal binary mixed homology systems C1_2_H_25_PO(CH_3_)_2_/C10H_21_PO(CH_3_)_2_, C_12_H_25_ (CH_3_)_3_NBr/C_16_H_33_ (CH_3_)_3_NBr in aqueous solutions (25 °C), C_8_F_15_O_2_NH_4_/C_9_F_17_O_2_NH4 in 0.1·mol·L^−1^ ammonium chloride solution (25 °C). Their research also suggested that the astringency velocity of the two kinds of iterative methods was very fast and the relative error of expression was below 1%. Wang *et al*. have defined synergisms in surface tension reduction efficiency and mixed micelle formation of binary surfactant mixtures in aqueous solution by appointing the ideal mixture system of surfactants as the standard of comparison, and they have deduced the conditions and the corresponding optimum point values of these two kinds of synergisms based on the regular solution theory and the ideal solution theory [104].

## 8. Conclusions and Prospects

In previous paragraphs, the progress in the QSPR studies on surfactants relating to cloud points, charge distribution, surface tension, degradation and other properties have been reviewed (Table 2). In earlier QSPR studies for surfactants cited in the present review, topological descriptors were mostly adopted to correlate activities/properties, however theses studies may lack insightfulness since there have been few investigations involving effects of electronic motion in molecules (or at intermolecular level) on their properties and functions that is highly critical to development and application of surfactants. Therefore quantum mechanical descriptors have been introduced to solve this problem. More recently, higher level of theory and larger basis sets have been used instead of semiempirical methods to get a higher accuracy for descriptors. For statistical methods, PLS, PCA and neural network are more and more improtant in this field for supplement of MLR. The model statistics developed by MLR are very often too optimistic, therefore the reliability of models should be checked by running for example leave-one-out, randomization or bootstrapping test. In addition, three-dimensional quantitative structure-property relationship (3D-QSPR) models can also be used to surfactants, especially in their biodegradation properties prediction.

At present, it has been understood that biological surfactants have some properties which synthetic surfactants do not have, especially in the aspects of biological degradation, safety and physiological activity. For surfactants or molecule with amphiphilic structure [105,106], effects of their structures on the functions mainly depend on various interfacial behavior and this is unavoidably related to intermolecular interactions between surfactant molecules and between surfactant molecules and between surfactant molecules and those of a solvent. Presently, for treatment of intermolecular interactions in surfactant solution, various approximate models based on classical electrostatic potential theory, such as multiple expansion approach (MPE), IC (image charge approximation) and apparent surface charge (ASC) approach, have been developed following continuum medium model [107]. In these models, using quantum mechanical ab initio and semiempirical methods, intermolecular interactions can be studied based on various cavity models and corresponding energies and charge distribution [108–110]. In addition, electrostatic interactions of charge fields force field calculations describing intermolecular interactions was used to correlate properties of small molecules and aggregates [111]. However, this method can only be applied to those small systems at this stage, and application of more advanced models to surfactants or molecules with amphiphilic to perform quantitative modeling still needs breakthrough in system interaction mechanism studies and theoretical calculations.

## Figures and Tables

**Table 1.** Results of the best four-parameter multilinear regression for cloud point of nonionic surfactants based on the 20-member training set.

**descriptor**	**No.**	**X**	**DX**	***t*-test**	***R* ^2^**	***F***	***S* ^2^**
intercept	0	–7.03E + 02	1.91E + 01	–36.7455			
relative molecular weight	1	1.77E + 02	4.33E + 00	40.9502	0.2983	8.08	313.55
average information content (order 2)	2	–4.53E + 01	2.56E + 00	–17.7164	0.6445	16.32	167.67
relative number of rings	3	–5.53E + 03	1.68E + 02	–32.8383	0.8516	32.51	74.14
Kier shape index (order 3)	4	–2.81E + 00	1.34E – 01	–21.0049	0.9948	765.99	2.75

X, regression coefficient; DX, error of the regression coefficient; *t*-test, value for the single regression term: *R*^2^, squared correlation coefficient for all regression terms just mentioned; *F,* value for the F-test for all regression terms just mentioned; *S*^2^, mean square deviation (variance) for all regression terms just mentioned.

**Table 2.** The reviewed contents.

1	**Introduction**
2	**Studies on Relationships between CMC And Molecular Structures**
2.1	*QSPR Studies on CMC of Surfactants Based on Molecular Connectivity Index*
2.2	*QSPR Studies of CMC of Surfactants Based on Quantum Mechanical Descriptors*
2.3	*QSPR Studies of CMC of Surfactants Using Neural Network*
2.4	*QSPR Studies of CMC of Surfactants Using Other Methods*
3	**Charge Distribution of Surfactants and Its Influence on Their Properties**
3.1	*Computation of Charge Distribution in Ionic Surfactant Molecules and Their Effects*
3.2	*Descriptors Related to Molecular Surface Area — CPSA*
4	**Surface Tension Prediction Models**
5	**QSAR Studies on Cloud Point of Nonionic Surfactants**
6	**Studies on Degradation of Surfactants**
7	**QSPR Studies on Other Properties of Surfactants**
8	**Conclusions and Prospects**

## References

[1] Li GZ, Sui H, Zhu WZ (1999). Progress of surfactant study [in Chinese]. China Surfact. Deterg. Cosm.

[2] William AE, the Committee on Fetus and Newborn (2008). Surfactant-replacement therapy for respiratory distress in the preterm and term neonate. Pediatrics.

[3] Riviere JE, Baynes RE, Xia XR (2007). Membrane-coated fiber array approach for predicting skin permeability of chemical mixtures from different vehicles. Toxicol. Sci.

[4] Wolf S, Lohbrunner H, Busch T, Sterner-Kock A, Deja M, Sarrafzadeh A, Neumann U, Kaisers U (2001). Small dose of exogenous surfactant combined with partial liquid ventilation in experimental acute lung injury: effects on gas exchange, haemodynamics, lung mechanics, and lung pathology. Br. J. Anaesth.

[5] Fendler JH (1987). Atomic and molecular clusters in membrane mimetic chemistry. Chem. Rev.

[6] Zhang XY, Zhu HW, Li L, Li GZ, Wang ZW (2003). The progress of qsar/qspr for surfactants [in Chinese]. Prog. Chem.

[7] Eibl H (1984). Phospholipids as functional constituents of biomembranes. Angew. Chem. Int. Ed. Engl.

[8] Jiang ZL, Xiao D, Wang KM, Xia SX (1995). Bilayer lipid membrane and biosensors [in Chinese]. Chem. Sensors.

[9] Crivori P, Morelli A, Pezzetta D, Rocchetti M, Poggesi I (2007). Development and validation of in silico models for estimating drug preformulation risk in PEG400/water and Tween80/water systems. Eur. J. Pharm. Sci.

[10] Stevens TP, Sinkin RA (2007). Surfactant replacement therapy. Chest.

[11] Zhang PF, Avudzega DM, Bowman RS (2007). Removal of perchlorate from contaminated waters using surfactant-modified zeolite. J. Environ. Qual.

[12] Wang LS (1991). Organic Pollutant Chemistry [in Chinese].

[13] Lehmler HJ (2005). Synthesis of environmentally relevant fluorinated surfactants—A review”. Chemosphere.

[14] Salager JL (2002). FIRP Booklet # 300-A: Surfactants-Types and Uses.

[15] Mason Chemical Company Fluorosurfactant—Structure/Function. Available online: http://www.masonsurfactants.com/Products/Fluorosurfactant.htm (accessed 1 November 2008).

[16] Kardanpour Z, Hemmateenejad B, Khayamian T (2005). Wavelet neural network-based QSPR for prediction of critical micelle concentration of Gemini surfactants. Anal. Chim. Acta.

[17] Zhao GX (1991). Physical Chemistry of Surfactants (revised edition) [in Chinese].

[18] Wang ZW, Feng JL, Wang HJ, Cui ZG, Li GZ (2005). Effectiveness of surface tension reduction by nonionic surfactants with quantitative structure-property relationship approach. J. Dispersion Sci. Technol.

[19] Katritzky AR, Pacureanu LM, Slavov SH, Dobchev DA, Karelson M (2008). QSPR study of critical micelle concentrations of nonionic surfactants. Ind. Eng. Chem. Res.

[20] Chen ML, Wang ZW, Wang HJ, Zhang GX, Tao FM (2007). Investigation of adsorption of surfactant at the air-water interface with quantum chemistry method. Chin. Sci. Bull.

[21] Wang ZW, Feng JL, Wang ZN, Li GZ, Lou AJ (2006). Quantitative structure-property relationship on prediction of the interaction parameters $\delta_{t}^{2}$ of organic compounds. J. Dispersion Sci. Technol.

[22] Wang LS, Han SK, Kong LR (1997). Molecular Structure, Properties and Activity [in Chinese].

[23] Katritzky AR, Lobanov VS, Karelson M (1995). QSPR-The correlation and quantitative prediction of chemical and physical properties from structure. Chem. Soc. Rev.

[24] Theil W, Voityuk AA (1996). Extension of MNDO to d-orbitals: parameters and results for the second-row elements and for the zinc group. J. Phys. Chem.

[25] Becher P (1984). Hydrophile lipophile balance: history and recent developments (Langmuir lecture, 1983). J. Dispersion Sci. Technol.

[26] Karelson M, Maran U, Wang YL, Katritzky AR (1999). QSPR and QSAR models derived using large molecular descriptor spaces. A review of CODESSA applications. Collec. Czechoslovak Chem. Commun.

[27] Wang ZW, Li GZ, Zhang XY, Li L (2002). Prediction on critical micelle concentration of anionic surfactants in aqueous solution: quantitative structure-property relationship approach [in Chinese]. Acta Chim. Sinica.

[28] Li L, Zhang XY, Wang ZW, Li GZ, Zhu HW, Zhang YS (2003). Study of quantitative structure-property relationship of nonionic surfactants [in Chinese]. China Surfact. Deterg. Cosm.

[29] Li XF, Zhang GY, Dong JF, Zhou XH, Yan XC, Luo MD (2004). Estimation of critical micelle concentration of anionic surfactants with QSPR approach. J. Mol. Struct.

[30] Huibers PDT, Lobanov VS, Katritzky AR, Shah DO, Karelson M (1996). Prediction of critical micelle concentration using a quantitative structure-property relationship approach: 1. nonionic surfactants. Langumir.

[31] Huibers PDT, Lobanov VS, Katritzky AR, Shah DO, Karelson M (1997). Prediction of critical micelle concentration using a quantitative structure property relationship approach: 2. anionic surfactants. J. Colloid Interface Sci.

[32] Kier LB, Hall LH (1986). Molecular Connectivity in Structure-activity Analysis.

[33] Stankevich MI, Stankevich IV, Zefirov NS (1988). Topological indices in organic chemistry. Russ. Chem. Rev.

[34] Stewart JJP MOPAC 6.0: A General Purpose Molecular Orbital PackageQCPE No. 455, Frank J. Seiler Research Laboratory, U.S. Air Force Academy Colorado Springs, CO, USA 1989

[35] Wang ZW, Li GZ, Zhang XY, Wang RK, Lou AJ (2002). A quantitative structure-property relationship study for the prediction of critical micelle concentration of nonionic surfactants. Colloids Surf. A: Physicochem. Eng. Aspects.

[36] Katritzky AR, Pacureanu L, Dobchev D, Karelson M (2007). QSPR study of critical micelle concentration of anionic surfactants using computational molecular descriptors. J. Chem. Inf. Model.

[37] Wang ZW, Huang DY, Gong SP, Li GZ (2003). Prediction on critical micelle concentration of nonionic surfactants in aqueous solution: Quantitative structure-property relationship approach. Chin. J. Chem.

[38] Wang ZN, Wang ZW, Gao YA, Zhen LQ, Liu J, Li GZ, Zhang GY (2004). QSPR cmc calculations for AE_3_SO_3_ and the contribution of EO in micellization [in Chinese]. Acta Chim. Sinica.

[39] Roberts DW (2002). Application of octanol/water partition coefficients in surfactant science: a quantitative structure-property relationship for micellization of anionic surfactants. Langmuir.

[40] Leo AJ, Hansch C (1979). Substituent Constants for Correlation Analysis in Chemistry and Biology.

[41] Roberts DW (1989). Aquatic toxicity of linear alkyl benzene sulphonates (LAS)-A QSAR approach. J. Com. Esp. Deterg.

[42] Puvvada S, Blankschtein D (1990). Molecular-thermodynamic approach to predict micellization, phase behavior, and phase separation of micellar solutions. I. Application to nonionic surfactants. J. Chem. Phys.

[43] Nagarajan R, Ruckenstein E (1991). Theory of surfactant self-assembly: a predictive molecular thermodynamics approach. Langumir.

[44] Zhang B, Liu HL, Hu Y (2001). Attraction between like-charge particles [in Chinese]. Prog. Chem.

[45] Huibers PDT (1999). Quantum-chemical calculations of the charge distribution in ionic surfactants. Langumir.

[46] Gadre SR, Pingale SS (1996). An electrostatic investigation: how polar are ionic surfactant hydrocarbon tails?. Chem. Commun.

[47] Jiang YB, Xu JG, Chen GZ (1991). Molecular structure of ionic surfactants in water solution. Sci. China Ser. B.

[48] Thiel W, Voityuk AA (1996). Extension of MNDO to d orbitals: parameters and results for the second-row elements and for the zinc group. J. Phys. Chem.

[49] Heidl A, Kohler HH (1996). Rod formation of ionic surfactants: a thermodynamic model. Langmuir.

[50] Zhao J, Fung BM (1993). NMR study of the transformation of sodium dodecyl sulfate micelles. Langmuir.

[51] Jacobs PT, Anacker EW (1973). The effect of polar head charge delocalization on micellar aggregation numbers of decylpyridinium salts. J. Collid Interface Sci.

[52] Huibers PDT, Jacobs PT (1998). The effect of polar head charge delocalization on micellar aggregation numbers of decylpyridinium salts, revisited. J. Colloid Interface Sci.

[53] Villamagna F, Whitehead MA, Chattopadyay AKJ (1995). A molecular modelling approach to the analysis of present and design of future surfactants for water-in-oil emulsions. J. Mol. Struct. (THEOCHEM).

[54] Camilleri P, Watts A, Boraston JA (1988). A surface area approach to determination of partition coefficients. J. Chem. Soc. Perkin Trans.

[55] Koehler MG, Grigoras S, Dunn WJ (1988). The Relationship between chemical structure and the logarithm of the partition coefficient. Quant. Struc.—Act. Relat.

[56] Grigoras S (1990). A structural approach to calculate physical properties of pure organic substances: the critical temperature, critical volume and related properties. J. Comput. Chem.

[57] Stanton DT, Jurs PC (1990). Development and use of charged partial surface area structural descriptors in computer-assisted quantitative structure-property relationship studies. Anal. Chem.

[58] Pearlman RS, Yalkowsky SH, Sinkula AA, Valvani SC, Marcel D (1980). Physical Chemical Properties of Drugs.

[59] Abraham RJ, Smith PE (1988). Charge calculations in molecular mechanics iv: A general method for conjugated systems. J. Comput. Chem.

[60] Wang ZW, Li GZ, Mu JH, Zhang XY, Lou AJ (2002). Quantitative structure-property relationship on prediction of surface tension of nonionic surfactants. Chin. Chem. Lett.

[61] Wang ZW, Feng JL, Wang HJ, Cui ZG, Li GZ (2005). Effectiveness of surface tension reduction by nonionic surfactants with quantitative structure-property relationship approach. J. Dispersion Sci. Technol.

[62] Wang ZW, Li GZ, Zhang XY, Liao LL Prediction on the effectiveness in surface tension reduction of anionic surfactants in aqueous solution: quantitative structure-property relationship approach.

[63] Wang ZW, Huang DY, Li GZ, Zhang XY, Liao LL (2003). Effectiveness of surface tension reduction by anionic surfactants, a quantitative structure-property relationship. J. Dispersion Sci. Technol.

[64] Stanton DT, Jurs PC, Hicks MG (1991). Computer-assisted prediction of normal boiling points of furans, tetrahydrofurans, and thiophenes. J. Chem. Inf. Comput. Sci.

[65] Stanton DT, Jurs PC (1992). Computer-assisted study of the relationship between molecular structure and surface tension of organic compounds. J. Chem. Inf. Comput. Sci.

[66] Ghasemi J, Ahmadi S (2007). Combination of genetic algorithm and partial least squares for cloud point prediction of nonionic surfactants from molecular structures. Anal. Chim.

[67] Chen ML, Wang ZW, Zhang GX, Gu J, Cun Z, Tao FM (2007). Studies on the cloud points of nonionic surfactants with QSPR. Chem. Res. Chinese U.

[68] Mitchell DJ, Tiddy GJT, Waring L, Bostock T, McDonald MP (1983). Phase behaviour of polyoxyethylene surfactants with water. J. Chem. Soc. Faraday Trans.

[69] Ren YY, Liu HX, Yao XJ, Liu MC, Hu ZD, Fan BT (2006). The accurate QSPR models for the prediction of nonionic surfactant cloud point. J. Colloid Interface Sci.

[70] Bünz AP, Braun B, Janowsky R (1998). Application of quantitative structure performance relationship and neural network models for the prediction of physical properties from molecular structure. Ind. Eng. Chem. Res.

[71] Huibers PDT, Shah DO, Katritzky AR (1997). Predicting surfactant cloud point from molecular structure. J. Colloid Interface Sci.

[72] Yuan SL, Cai ZT, Xu GY, Wang W (2003). Prediction of cloud poiont of nonionic surfactants using quantitative structure-property relationship method. Acta Phys. Chim. Sin.

[73] Guan JQ, Li JS (1994). Biodegradation of surfactants in the environment [in Chinese]. Environ. Sci.

[74] Schöbel P, Marl FRG (1989). Basic principles of LAS degradation. Tenside Surfactants Detergents.

[75] Qu FP, Yang YY, Feng XD, Qi J, Dai YY (1999). Research principle and progress of QSBR (Quantitative Structure Biodegradability Relationships) [in Chinese]. China Environ. Sci.

[76] European Parliament Regulation (EC) No 648/2004 of the European Parliament and of the Council of 31 March 2004 on detergents. EC No 684/2004. In *Official Journal of the European Union*, published in Luxembourg, 8 April 2004.

[77] Merrettig-Bruns U, Jelen E (2009). Anaerobic biodegradation of detergent surfactants. Materials.

[78] Siwiski P, Szymaski A, Lukaszewski Z (1998). Biodegradability of detergent powder surfactants in the river water die-away test. Polish J. Environ. Stud.

[79] Sales D, Perales JA, Manzano MA, Quiroga JM (1999). Anionic surfactant biodegradation in seawater. Bol. Inst. Esp. Oceanogr.

[80] Li L, Zhang XY, Wang ZW, Li GZ (2003). Structure-biodegradation relationship study of commercial linear alkylbenzene sulfonates. Internet Electr. J. Mol. Des.

[81] Schultz MM, Barofsky DF, Field JA (2003). Fluorinated alkyl surfactants. Environ. Eng. Sci.

[82] Nabb DL, Szostek B, Himmelstein MW, Mawn MP, Gargas ML, Sweeney LM, Stadler JC, Buck RC, Fasano WJ (2007). *In Vitro* metabolism of 8-2 fluorotelomer alcohol: interspecies comparisons and metabolic pathway refinement. Toxicol. Sci.

[83] Parsons JR, Sáez M, Dolfing J, Voogt P (2008). Biodegradation of perfluorinated compounds. Rev. Environ. Contam. Toxicol.

[84] Guruge KS, Yeung LWY, Yamanaka N, Miyazaki S, Lam PKS, Giesy JP, Jones PD, Yamashita N (2006). Gene expression profiles in rat liver treated with perfluorooctanoic acid (PFOA). Toxicol. Sci.

[85] Dimitrov S, Kamenska V, Walker JD, Windle W, Purdy R, Lewis M, Mekenyan O (2004). Predicting the biodegradation products of perfluorinated chemicals using catabol. SAR QSAR Environ. Res.

[86] Fei CY, McLaughlin JK, Lipworth L, Olsen J (2009). Maternal levels of perfluorinated chemicals and subfecundity. Hum. Reprod.

[87] Dimitrov S, Kamenska V, Walker JD, Windle W, Purdy R, Lewis M, Mekenyan O (2004). Predicting the biodegradation products of perfluorinated chemicals using catabol. SAR QSAR Environ. Res.

[88] Torres FJ, Ochoa-Herrera V, Blowers P, Sierra-Alvarez R (2009). *Ab initio* study of the structural, electronic, and thermodynamic properties of linear perfluorooctane sulfonate (PFOS) and its branched isomers. Chemosphere.

[89] Chen ML, Wang ZW, Zhang GX, Wang WD, Wang ZN (2007). Prediction on hydrophile-lipophile balance values of anionic surfactants with QSPR method [in Chinese]. Acta Chim. Sinica.

[90] Davies JA, Hockensmith CM, Kukushkin VY, Kukushkin YN (1996). Synthetic Coordination Chemistry.

[91] Liu SL, Tong JB, Li YF (2009). QSPR Study on hydrophile-lipophile balance values of anionic surfactant [in Chinese]. Guangdong Chem.

[92] Ghasemi J, Abdolmaleki A, Asadpour S, Shiri F (2008). Prediction of solubility of nonionic solutes in anionic micelle (SDS) using a QSPR model. QSAR Comb. Sci.

[93] Campbell P, Strinivasan R, Knoell T, Phipps D, Ishida K, Safarik J (1999). Quantitative structure-activity relationship (QSAR) analysis of surfactants influencing attachment of a Mycobacterium sp. to cellulose acetate and aromatic polyamide reverse osmosis membranes. Biotechnol. Bioeng.

[94] Biz S, Occelli ML (1998). Synthesis and characterization of mesostructured material. Catal. Rev. Sci. Eng.

[95] Missel PJ, Mazer NA, Benedek GB, Young CY, Carey MC (1980). Thermodynamic analysis of the growth of sodium dodecyl sulfate micelles. J. Phys. Chem.

[96] You H, Zhao B, Wang ZW (2009). Mesoscopic simulation on phase behavior of sodium polyoxyethylene fatty alcohol sulfate in aqueous solution. Acta Phys.—Chim.

[97] Lindgren A, Sjostrom M, Wold S (1996). Quantitative-structure-effect relationship for some technical nonionic surfactants. J. Amer. Oil Chem. Soc.

[98] Warszynski P, Lunkenheimer K (1999). Influence of conformational free energy of hydrocarbon chains on adsorption of nonionic surfactants at the air/solution interface. J. Phys. Chem. B.

[99] Wang ZW, Yi XZ (2007). Calculation of the molecular exchanging energy of binary surfactants system on the surface monolayer of aqueous solution. Sci. China Ser. B.

[100] Vitha MF, Carr PW (1998). A linear solvation energy relationship study of the effects of surfactant chain length on the chemical interactions governing retention and selectivity in micellar electrokinetic capillary chromatography using sodium alkyl sulfate buffers. Sep. Sci. Technol.

[101] Rosen MJ (1987). Surfactants and Interfacial Phenomena.

[102] Andreea PB, Muriel B, Maura B, Emile P, Christophe M, Isabelle RL, Thomas L, Reiko O (2005). Spontaneous vesicles of single-chain sugar-based fluorocarbon surfactants. J. Fluor. Chem.

[103] Tu GY, Wang ZW, Wang ZN, Liu F, Xiao JY (2008). Theoretical expression and experiment on surface tension in ideal binary mixtures of surfactants [in Chinese]. Acta Phys.—Chim. Sin.

[104] Wang ZW, Yu HX, Wang ZN, Li GZ (2006). Theoretical further study on synergisms of binary surfactant mixtures in aqueous solution. J. Dispersion Sci. Technol.

[105] Lü Q, Gong HF, Liu MH (2001). Progress in bolaamphiphile [in Chinese]. Prog. Chem.

[106] McGregor C, Perrin C, Monck M, Camilleri P, Kirby AJ (2001). Rational approaches to the design of cationic gemini surfactants for gene deliver. J. Am. Chem. Soc.

[107] Tomasi J, Persico M (1994). Molecular interactions in solution: An overview of methods based on continuous distributions of the solvent jacopo tomasi, maurizio persico. Chem. Rev.

[108] Karelson MM, Zerner MC (1992). Theoretical treatment of solvent effects on electronic spectroscopy. J. Phys. Chem.

[109] Szafran M, Karelson MM, Katritzky AR, Koput J, Zerner MC (1993). Reconsideration of solvent effects calculated by semiempirical quantum chemical methods. J. Comput. Chem.

[110] Wong MW, Frisch MJ, Wiberg KB (1991). Solvent effects. 1. The mediation of electrostatic effects by solvents. J. Am. Chem. Soc.

[111] Dykstra CE (1993). Electrostatic interaction potentials in molecular force fields. Chem. Rev.

